# Macroporous Alginate–PEG Hybrid Double Network Cryogels: Tuning Mechanics, Porosity, and Long-Term Growth Factor Release via Polymer Concentration, Ice Nucleation, and Sulfation

**DOI:** 10.1021/acsabm.5c01929

**Published:** 2025-12-27

**Authors:** Zining Yang, Kaixiang Zhang, Michael Patrick Seitz, Era Jain

**Affiliations:** † Department of Biomedical and Chemical Engineering, 2029 Syracuse University, Syracuse, New York 13244, United States; ‡ Bioinspired Syracuse Institute for Material and Living System, Syracuse University, Syracuse, New York 13244, United States

**Keywords:** cryogel, tissue engineering, growth factors, scaffold, click chemistry

## Abstract

Cryogels are macroporous hydrogel scaffolds with promising applications in tissue engineering; however, conventional systems often suffer from inadequate mechanical strength, limited porosity, and biocompatibility concerns due to the use of free radical initiators and organic solvents. In this study, we report the engineering of a hybrid double-network (HDN) cryogel scaffold composed of alginate and poly­(ethylene glycol) (PEG), designed for tissue engineering applications. The scaffolds were fabricated via simultaneous cross-linking of alginate and PEG networks at subzero temperatures using a solvent-free and initiator-free biocompatible approach. We systematically investigated the influence of polymer concentration, ice nucleating agents, and sulfation of alginate on the resulting cryogel structure, porosity, and mechanical properties. The optimized HDN cryogels exhibited a highly interconnected macroporous architecture, improved compressive strength, and enhanced elasticity while maintaining cytocompatibility. These findings highlight the potential of HDN cryogels as robust and biocompatible scaffolds for soft tissue engineering and regenerative medicine applications.

## Graphical abstract

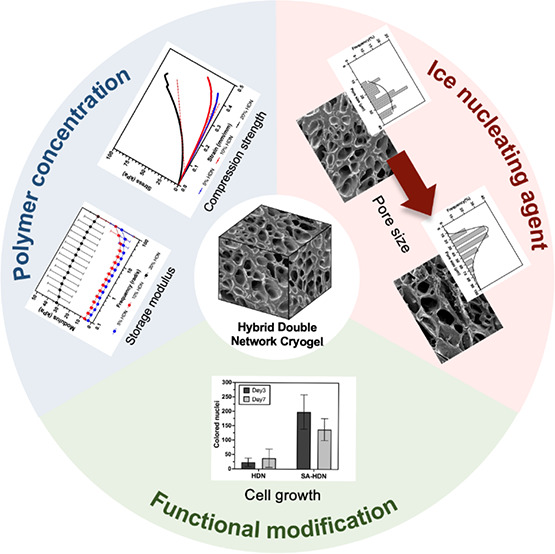

## 1 Introduction

Cryogels represent a distinct class of macroporous hydrogels formed through polymerization or gelation of gel forming precursors at temperatures below the freezing point of the aqueous solvent. Gelation at subzero temperatures leads to freezing of most of the solvent, leaving behind a small volume of nonfrozen aqueous liquid phase (NFLP) where most of the gel forming precursors are concentrated. The cryo-concentration of polymeric precursors around ice crystals results in formation of thick polymeric walls and a web of interconnected macropores upon thawing and melting away of ice crystals.^1–8^ Compared to conventional hydrogels prepared at room temperature, cryogels can support enhanced cell infiltration, tissue integration, and superior biological performance due to improved oxygen, nutrient, and waste exchange through the interconnected macroporous structure. The unique fabrication method imparts cryogels with remarkable mechanical stability, elasticity, high water uptake capacity, and high permeability, making them ideal for a variety of biomedical applications, including tissue engineering and drug delivery.^2,4,6,9–12^

We recently reported the synthesis of a biodegradable PEG-alginate hybrid double network (HDN) cryogel. The hybrid double network cryogel made using a radical-free cross-linking reaction chemistry allows customized degradation rate. This radical-free cross-linking strategy avoids organic solvents and initiators while enabling tunable degradation profiles based on the chemical identity of the PEG cross-linker. The interpenetrating double-network architecture combines the biocompatibility of alginate with the mechanical reinforcement provided by PEG, yielding scaffolds that are robust yet compliant in dynamic biological environments.^13^ Importantly, this approach does not require chemical modification of alginate or the use of high concentrations that often result in highly viscous, difficult-to-process solutions, while lower concentrations typically fail to form stable gels.^14,15^

The structural and functional properties of cryogels are strongly influenced by polymer concentration, freezing conditions, and polymer architecture.^16–18^ High polymer concentrations lead to denser, mechanically stronger networks but reduce pore size, whereas lower concentrations produce larger pores at the expense of strength.^19–23^

Similarly, lower synthesis temperatures generally yield smaller pores; however, excessive supercooling produces irregular ice crystals that reduce porosity, impede mass transport, and hinder reproducibility.^24–29^ In addition, sluggish cross-linking kinetics at very low temperatures further compromise gel formation. Incorporation of ice nucleating agents (INAs) has been proposed to overcome these limitations by promoting controlled ice nucleation, thereby enhancing pore uniformity and improving cryogel reproducibility. For example, Kressler and colleagues demonstrated that amino acids such as l-aspartic acid mitigate supercooling effects in poly­(vinyl alcohol) cryogels.^30,31^ Despite these promising findings, the use of ice nucleating agents has not yet been systematically explored in alginate- or PEG-based cryogels. Thus, the use of ice nucleating agents should be optimized to maintain an appropriate balance between porosity and mechanical integrity, ensuring the intended functional properties of the cryogels.

Alginate is widely used for tissue engineering scaffolds due to its similarity in structure with glycosaminoglycans (GAGs), an important component of the natural extracellular matrix of the cells. To further improve functionality, sulfated alginate offers additional advantages. As an analog of GAGs, sulfated alginate carries negatively charged sulfate groups similar to those in heparan sulfate compared with unmodified alginate to bind heparin-binding growth factors such as TGF-β1.^32^ The incorporation of sulfated alginate better mimics the native ECM in the cartilage and other tissues, enhances growth factor sequestration, and enables controlled release kinetics, making it a highly promising component for the design of bioactive and regenerative biomaterials.^33^ Thus, incorporation of sulfated alginate can potentially address one of the major limitations of macroporous scaffolds—rapid growth factor release due to high surface area and unrestricted diffusion. Incorporating sulfated alginate into HDN cryogels could therefore significantly broaden their utility for regenerative medicine.^34–36^ Thus, investigating the impact of sulfated alginate on cryogel properties will further enhance their application for controlled protein release and tissue engineering.

We previously reported PEG–alginate hybrid double-network cryogels that emphasized degradation control through regulation of the chemical identity of the cross-linker. In this study, we build upon our framework of click chemistry and ionic cross-linking for degradable, macroporous PEG–alginate HDN cryogels and expanded the design space for engineering cryogels with tunable physical and biological properties. We systematically investigated three new critical parameters: (i) varying the PEG network content (5%, 10%, 20% w/v), (ii) incorporating ice nucleating agents (l-aspartic acid), and (iii) applying sulfated alginate (SA-alginate) to enhance structure and performance. This systematic exploration highlights how a double-network architecture, controlled pore formation, and ECM-mimetic functionalization can be leveraged to engineer cryogels with optimized porosity, strength, and biological performance. These insights further expand the tunability of our cryogel system for targeted applications, including soft tissue engineering, regenerative medicine, and controlled drug delivery.

## 2 Materials and Methods

### 2.1 Materials

Sodium alginate (GMB, Manugel), formamide (≥99.5%, Sigma-Aldrich, St Louis, MO, USA), chlorosulfonic acid (HClSO_3_, 99%, Sigma-Aldrich, St Louis, MO, USA), acetone (99.5%, Acros Organics, Geel, Belgium), sodium hydroxide (NaOH, 8M, Honeywell-Fluka, Charlotte, North Carolina, USA), sodium chloride (NaCl, ThermoFisher, Waltham, MA, USA), deuterium oxide (D_2_O, 99.9%, Cambridge Isotope Labratories, Inc., Tewksbury, MA, US), 8-arm-PEG-acrylate (8-arm PEGAc,10 kDa, Jenkem, Plano, TX, USA), (S)-2-aminobutane-1,4-dithiol (DTBA, 99%, Sigma-Aldrich, St Louis, MO, USA), calcium carbonate (CaCO_3_, 99+%, Acros Organics, Geel, Belgium), glucono-delta-lactone (GDL, 99%, Acros Organics, Geel, Belgium), *N*-2-hydroxyethylpiperazine-*N*-2-ethanesulfonic acid (HEPES, ThermoFisher, Waltham, MA, USA), Dulbecco’s phosphate-buffered saline (DPBS, Gibco, Waltham, MA, USA), DMEM/F12 (1:1) (with l-Glutamine and 15 mM Hepes, Gibco, Waltham, MA, USA), fetal bovine serum (FBS, Gibco, Waltham, MA, USA), penicillin-streptomycin (PS, Gibco, Waltham, MA, USA), transforming growth factor-beta 1 (TGF-β1, Invitrogen, Waltham, MA, USA), insulin-like growth factor 1 (IGF-1, PeproTech, Cranbury, NJ, USA), trypsin-EDTA (0.05%, Gibco, Waltham, MA, USA), collagen I (rat tail, Gibco, Waltham, MA, USA), acetic acid (ThermoFisher, Waltham, MA, USA), ethidium homodimer (2 mM, ThermoFisher, Waltham, MA, USA), calcein AM (BD Biosciences, Franklin Lakes, NJ, USA), Leibovitz’s (L-15 medium, Gibco, Waltham, MA, USA), 4’,6-diamidino-2-phenylindole (DAPI, ThermoFisher, Waltham, MA, USA), and paraformaldehyde (PFA, 4%, ThermoFisher, Waltham, MA, USA) were used.

### 2.2 Sulfation of Alginate

Alginate (100 mg) was suspended in 3.92 mL of formamide, and the mixture was stirred overnight at 60 °C. The alginate-formamide mixture was then added dropwise to 80 μL of concentrated HClSO_3_ (99%), resulting in a 2% v/v concentration of HClSO_3_, and stirred for 2.5 h at 60 °C. Two ml of cold acetone was added to the solution at room temperature (RT). After being vortexed for at least 3 min to ensure complete dissolution, the final solution was centrifuged at 5000 rpm for 7 min, and the precipitate was filtered by filter paper. Then, the precipitate was dissolved in 3–4 mL of deionized (DI) water and stirred for 2–3 h for complete dissolution with the help of NaOH. The solution was dialyzed against 1 L of 75 mM sodium chloride (NaCl) for 1 day, and then against DI water for 2 days. The NaCl solution and DI water were changed every 12 h. Finally, the dialyzed solution was frozen and then lyophilized to obtain sulfated alginate. The sulfation of alginate was validated by Fourier Transform Infrared (FTIR) and Nuclear Magnetic Resonance (NMR) spectroscopy. For the FTIR analysis (Thermo IS5, Waltham, MA, USA), dehydrated sulfated alginate was placed on a crystal plate. The spectrum was collected between 400 and 4000 cm^–1^ at a resolution of 16 cm^–1^ and 64 scans per spectrum. Sodium alginate was tested as a control. For NMR analysis, sulfated alginate was dissolved in deuterium oxide (D_2_O) at ∼7 mg/mL concentration. 1H NMR experiments were recorded at 25 °C on a 400 MHz Avance III HD Spectrometer (Bruker, Billerica, USA).

### 2.3 Synthesis of Cryogels

Hybrid double-network cryogels were synthesized through the simultaneous gelation of an alginate network and an 8-arm PEGAc network. First, 1.25% w/v alginate stock solution was prepared in HEPES buffer (0.1 M, pH 7.4) at room temperature by dissolving alginate in the buffer for at least 1 h. 8-arm PEGAc was then added at the final concentration of 5, 10, and 20% w/v to the alginate solution. 8-arm PEGAc-alginate solution mix was vortexed for 10 s and centrifuged at 4000 rpm for 5 min to remove any bubbles. 0.3 M calcium carbonate, 0.6 M glucono-delta-lactone (GDL), and 0.8 M DTBA were dissolved in HEPES buffer (0.01 M, pH 7.4) to get a 100 μL 10× DTBA stock solution. Both 8-arm PEGAc-alginate solution and 10× cross-linker stock solution were cooled to 4 °C on ice, and then 10× cross-linker stock solution was added to 8-arm PEGAc-alginate solution. The 8-arm PEG network of cryogels was produced via a Michael addition reaction between acrylate and thiol groups. The final concentration of alginate in the cryogel precursor solution was 1% w/v, and 8-arm PEGAc network varied between 5%, 10%, and 20% w/v. All mixtures were immediately stirred on the vortex mixer for 15 s and incubated for at least 18 h in the refrigerated circulating bath (VWR, PA, USA) maintained at −20 °C. The cryogels were removed from the freezing conditions and immersed in deionized water at RT for 15 min. Further, the cryogels were washed in DI water, dried using a Freeze-Dryer, and stored for further experiments.

For cryogels containing sulfated alginate, the alginate was replaced with 1% w/v sulfated alginate, while the rest of the procedure for cryogel synthesis was the same as described above.

To observe the effect of ice nucleating agents, l-aspartic acid was added at a concentration of 1% to 2% w/v into 20% w/v PEG-Ac HDN cryogels as an ice nucleating agent during fabrication. Since ice nucleating agents may elevate the ice nucleation temperature, cryogels containing l-aspartic acid were prepared at −12 °C and −20 °C to isolate temperature effects from compositional effects. The remaining procedure for cryogel fabrication was the same as described above.

### 2.4 Swelling Capacity Measurements

For swelling capacity, all cryogels were measured using a gravimetric method. The cryogel samples were standardized to uniform dimensions, each measuring 6 mm in both diameter and height. The cryogels were dried in an alcohol gradient (20%, 40%, 60%, 80%, and 100%), and the initial dry weight (Wi) was recorded. Dried cryogels were placed in PBS buffer (supplemented with 2 mM calcium chloride, pH 7.4) and incubated at 37 °C with gentle rocking. The weight of the swollen cryogels (Ws) was measured at regular time intervals. Before each measurement, excessive water on the cryogels’ surface was wiped off with a KimWipe. All samples were tested in triplicates. The swelling capacity of the cryogels was determined using the two parameters of the water uptake capacity and the equilibrium swelling ratio using the following equations:

1 Wateruptake(%)=(Ws−Wi)/Wi×100%

2 Equilibriumswellingratio:Qm=Ws/Wi

### 2.5 Scanning Electron Microscopy

The surface morphology of the cryogels was observed using scanning electron microscopy (SEM; JEOL JSM 5600, Akishima, Japan). Before the measurement, the cryogels were cut into 5 mm height discs, dried in an alcohol gradient, and coated with gold by a sputter coater (Desk V, Denton Vacuum, Moorestown, USA). For each group, at least 30 pores were measured from five randomly selected micrographs collected from three independent cryogel samples, and the obtained diameters were grouped into 15 μm intervals to generate pore size distribution histograms. The average pore diameters were determined by using threshold and “measure particles” functions in ImageJ.

### 2.6 Rheological Measurements of Cryogels

Rheological measurements were conducted on a TA-DHR3 rotational rheometer (TA Instruments, New Castle, USA). For rheology testing, all cryogels were (diameter 8 mm and height ∼6 mm) incubated in 1× PBS (with 2 mM calcium chloride, pH 7.4) buffer for 2 h at 37 °C. Before each measurement, the cryogel surface was gently wiped off by KimWipe to blot any excess water. A constant frequency of 1 rad/s and a strain range of 0.01–1% were used for the strain amplitude tests. A constant strain of 0.01% and a frequency range of 0.1–100 rad/s were used for the frequency sweep tests. All tests were conducted in triplicate. The storage and loss moduli were expressed as the average ± standard deviation.

### 2.7 Compression Analysis of Cryogels

The compression test of cryogels was conducted using an Electromechanical Universal Testing Machine (Shimadzu, Kyoto, Japan) and recorded by TRAPEZIUM X (Shimadzu, Kyoto, Japan). Cryogels with a diameter of 8 mm and a height of ∼6 mm were swollen to equilibrium and positioned between the two flat plates of the load frame. A preload of 0.1 N was applied to maintain firm contact between the plates and the sample. The cryogel samples were compressed to 70% of their original length at a speed of 1 mm/min with the 100 N load cell. Both the compression force and the change in column length as a result of compression were recorded. The following equations were used to calculate stress, strain, and compression modulus of the cryogels:

3 Strain=ΔL/L

4 Stress=F/A

5 E=(F/A)/(ΔL/L)

(Δ*L*: change in height; *L*: height of sample; *F*: force applied on sample; *A*: cross-sectional area of the sample; *E*: Young’s modulus of elasticity).

The linear region of the stress-strain curves was analyzed to determine the compression modulus. Three samples for each cryogel were used for testing, and the compression modulus was reported as average ± standard deviation.

### 2.8 Cell Culture in Cryogels and Cell Viability Analysis

Mouse mesenchymal stem cells (MSCs), D1 cells, were cultured in DMEM F12 media supplemented with 10% FBS and 1% PS, in a humidified 5% CO_2_ incubator at 37 °C. Upon reaching 80% confluence, cells were detached from the bottom of the flask after incubation with 0.25% trypsin and 0.05% EDTA for 5 min at 37 °C and centrifuged to obtain a cell pellet. The harvested cells were resuspended in fresh DMEM F12 medium to achieve a concentration of 3.3 × 10^6^ cells/ml.

Before cell seeding, cryogels were cut to a diameter of 4 mm and a height of ∼1.5 mm and sterilized with 70% ethanol. Subsequently, they were coated with collagen type I (50 μg/mL) to enhance cell adhesion. The cryogels were partially dehydrated under sterile conditions for 3 h.

For cell seeding, 30 μL of cell suspension containing 2 × 10^5^ cells was applied to the surface of each dehydrated cryogel and allowed to attach to the cryogels for 1 h before adding media. The cell-loaded cryogels were incubated in 200 μL of DMEM F12 medium at 37 °C with 5% CO_2_. DMEM media was refreshed every 2 days.

### 2.9 Cell Viability Measurement

To assess the biocompatibility of cryogels, the cell viability was determined using a live/dead assay. After MSCs D1 cells were cultured for 3 or 7 days, DMEM media was removed, and the cell-loaded cryogel scaffold was washed three times with PBS buffer to remove any unattached cells. The samples were then incubated with Calcein-AM (green) and ethidium homodimer (red) for 30 min, as per the manufacturer protocol (LIVE/DEAD viability/cytotoxicity kit, Invitrogen, Carlsbad, USA). Additionally, DAPI staining was performed for cell viability evaluation. Briefly, cell-loaded cryogels were incubated with 300 nM DAPI for 45 min after being fixed with 4% PFA.

Following the washing off of the excess dye, fluorescence images were captured using a Thunder upright microscope (Leica, Wetzlar, Germany) and analyzed using ImageJ software to calculate the ratio of live cells to dead cells.

### 2.10 Growth Factor Loading and Release Kinetics Measurement

Dehydrated cryogels (∼4 mm in diameter and 1.5 mm in height) were soaked in DMEM-F12 media containing TGF-β1 (30 μL, 3.33 ng/mL) or IGF-1 (30 μL, 3.33 ng/mL). Following two overnight incubations, the remaining medium was collected, and samples were washed once. The samples were then placed in 200 μL of DMEM-F12 medium containing 1% penicillin-streptomycin (PS) and incubated on a shaker at 37 °C. The medium in the tubes was collected regularly and replaced with 200 μL of fresh medium. The released amounts of TGF-β1 and IGF-1 were quantified using standard ELISA, following the manufacturer’s instructions. The total amount of growth factor loading was determined by subtracting the unabsorbed growth factor fraction in the soaking medium and the initial wash as determined by ELISA.

#### 2.10.1 Model Fitting of Kinetics of Growth Factor Release

The release profiles of TGF-β1 and IGF-1 from 20% HDN and SA-HDN cryogels were analyzed using mathematical models frequently applied to characterize drug release mechanisms from polymeric matrices.^37,38^ The following kinetic models were evaluated:

**Zero-order model:**

6 $$\frac{M_{t}}{M_{\infty }}=K_{0}t$$

**First-order model:**

7 $$\frac{M_{t}}{M_{\infty }}=1-e^{-k_{1}t}$$

**Korsmeyer–Peppas (KP) model:**

8 $$\frac{M_{t}}{M_{\infty }}=k_{2}t^{n}$$

The KP model is widely used for polymeric delivery systems including hydrogels. It is particularly useful when the release mechanism is complex or when multiple processes contribute to release. The diffusional exponent **n** allows classification of the dominant mechanism, distinguishing between Fickian diffusion and anomalous transport.

**Higuchi model:**

9 $$\frac{M_{t}}{M_{\infty }}=k_{H}t^{1/2}$$

In all equations, *M*_t_ represents the cumulative amount of growth factor released at time *t*, *M*_∞_ represents the amount released at infinite time, *k*is the kinetic rate constant for each respective model, and *n*is the KP diffusional exponent describing the release mechanism.

For each model, the cumulative fraction of growth factor released was plotted as a function of time. Parameter estimation and curve fitting were performed by using the Solver function in Microsoft Excel. The goodness of fit was assessed using the coefficient of determination (**R** ^**2**^) obtained from regression analysis. The model exhibiting the highest R^2^ value for each data set was considered the most appropriate to describe the release mechanism from the cryogels.

### 2.11 Statistical Analysis

GraphPad Prism and Origin were used to conduct statistical analysis. Each experiment was conducted using at least 3 replicates unless stated otherwise. Results are expressed as average ± standard deviation. For comparison between groups, a *t* test or one-way ANOVA was used with a post hoc test. A *p*-value of <0.05 was considered statistically significant.

## 3 Results and Discussion

### 3.1 Overview and Preparation of Cryogels

In this study, we synthesized PEG-alginate HDN cryogels by combining alginate and multiarm PEG acrylate using a biocompatible chemical scheme and freezing them at subzero temperature. Based on previous studies, we used −20 °C as our optimum temperature for synthesis of cryogels to balance the reaction progression, cryogels synthesis, and ice nucleation for optimum pore size.^13^ Further, we used 50 mM HEPES buffer at pH 7.4 for synthesis of the PEG network and to facilitate efficient Michael addition between multiarm PEG acrylate and the thiol cross-linker. Thiol–Michael addition between multiarm PEG acrylate and the dithiol cross-linker is base-catalyzed since thiolate is the reactive species. At pH 7.4, thiols are deprotonated to thiolate for cryogelation. Such pH value ensures sufficient thiolate formation to enable the Michael-type cross-linking between multiarm PEG acrylate and dithiols. Previous work by us investigated the effect of pH on thiol cross-linking in the hydrogels formed by the Michael addition reaction between thiol and acrylate.^39^ We and others have shown that the reaction proceeds with high efficiency at pH 7.4, and reaction efficiency increases with increasing pH.

We systematically varied each of the three parameters (Figure 1) and investigated the effect of these variations on the physical properties of the cryogel. Table 1 presents the different parameters we used in this study. We investigated 8-arm-PEGAc-DTBA-alginate HDN cryogels with PEG polymer concentrations of 5%–20% w/v. This range was selected because cryogels synthesized at lower concentrations either exhibited inadequate mechanical integrity or did not form at all, while at PEG concentration higher than 20%, the gel precursor solution became too viscous and was difficult to work with and pour into desired molds.

**Figure 1:**
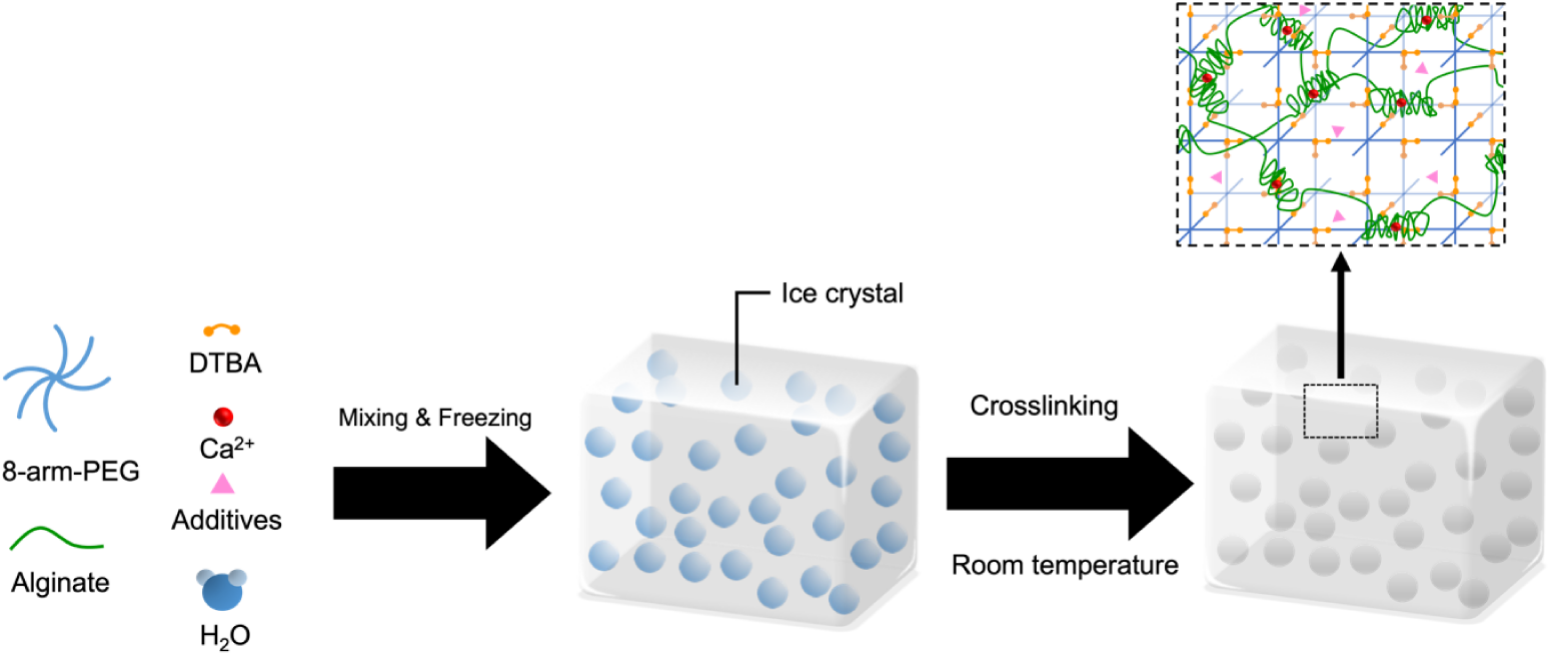
Schematic of PEG-alginate hybrid double-network cryogels formation. Alginate and the cross-linker calcium carbonate, 8-arm-PEGAc, and the dithiol cross-linker DTBA were mixed in an aqueous buffer and incubated at subzero temperatures. Ice crystals formed in the precursor solution upon freezing. After gelation at subzero temperatures for over 19 h, the mixture was thawed at room temperature. The ice crystals melted away, leaving behind interconnected macroporous cryogels.

**Table 1:** Different Parameters for Cryogel Synthesis Investigated in This Study

**Cryogel Components**				
**Alginate** **(1**% **w/v)**	**8-arm PEG acrylate %** **(w/v)**	**Abbreviated name**	l **-aspartic acid**	**Incubation temperature (°C)**
Alginate	20%	20% HDN	No	–20
Alginate	5%	5% HDN	No	–20
Alginate	10%	10% HDN	No	–20
Alginate	20%	1% AA-HDN	1%	–12
Alginate	20%	2% AA-HDN	2%	–12
Sulfated alginate	20%	SA-HDN	No	–20

Additionally, we examined the effects of using l-aspartic acid (Asp), an ice-nucleating agent, on the properties of PEG-alginate HDN cryogels. When prepared under the same freezing temperature as other cryogel formulations (−20 °C), 20% HDN cryogels containing Asp exhibited even smaller pore sizes than 20% HDN cryogels without Asp, which could be attributed to the promotion of rapid and extensive ice nucleation by Asp, leading to the formation of a larger number of smaller ice crystals (Figure S1). We then reduced the freezing temperature to −12 °C and found that 20% HDN cryogels containing Asp (1% AA-HDN) prepared at this higher temperature displayed higher porosity and larger pore sizes compared with those prepared at −20 °C. Thus, we used −12 °C as the incubation temperature for 20% HDN cryogels with l-aspartic acid.

Further in an attempt to mimic the natural ECM properties and its affinity for cells, we modified the alginate by introduction of the sulfate groups.^40,41^ To introduce sulfate groups, hydroxyl groups on the alginate backbone, primarily at the C-2 position of the sugar monomers, were sulfated by reaction with HClSO_3_ in formamide.

The FTIR spectrum of sulfated alginate (Figure 2A) showed a major peak at 1250 cm^–1^ and a minor peak at 800 cm^–1^ while these peaks were absent in the sodium alginate spectrum. The peak observed at ∼1250 cm^–1^ corresponds to the symmetric stretching of the S=O bond, while the peak at ∼800 cm^–1^ corresponds to the stretching of the S–O–C bond.^42^ Thus, the presence of these peaks indicates successful sulfation of the sodium alginate. 1H NMR analysis further confirmed the sulfation of the alginate (Figure 2). After sulfation, the proton signal near 5.0 ppm shifted significantly downfield and changed its peak shape. The multiplets in the 4.0–4.6 ppm region shifted toward higher chemical shifts and became more complex. Furthermore, a new signal appeared at ∼2.5 ppm. These changes are attributed to the strong electron-withdrawing effect of the sulfate group, which alters the electronic environment of protons adjacent to the sugar ring. This evidence suggests the introduction of new chemical groups into the alginate chain.^43,44^ Modified sulfated alginate was used in the preparation of PEG-alginate HDN cryogels.

**Figure 2:**
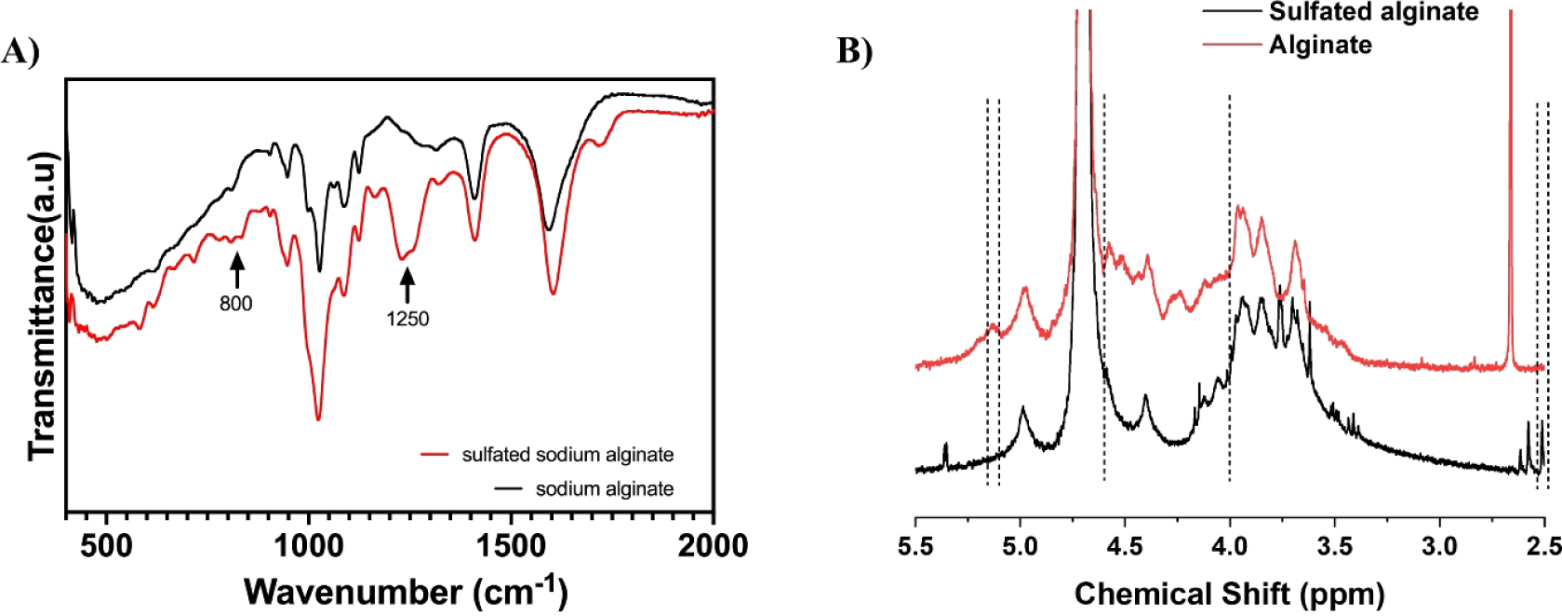
Spectroscopic verification of chemical modification of alginate by introduction of a sulfate group. (A) FTIR spectra of sulfated alginate and alginate. The arrows indicate observed peaks at 800 cm^–1^ the 1250 cm^–1^, which correspond to the S–O–C stretching and the S=spectra of O symmetric stretching, respectively, indicating the successful sulfation of the alginate. (B) ^1^H NMR spectra of sulfated alginate and alginate.

### 3.2 Morphological Analysis of Cryogels

Cryogelation involves freezing a polymer solution or suspension, allowing it to form a gel, and subsequently eliminating the ice crystals to create a porous structure. The size and arrangement of ice crystals formed during cryogelation are the primary factors that determine the surface morphology and pore size of the cryogel.^9,17^ Figure 3 shows the surface morphology and pore characteristics of cryogels made by varying different parameters during the synthesis. Notably, the pore size obtained from SEM images represents the apparent pore diameter of the freeze-dried cryogels, which may differ from the hydrated state but allows comparison among the groups prepared under the same conditions.^45,46^ Based on SEM images, the pore size distribution of each cryogel group was quantified (Figure 4). Among the cryogels with varying PEG concentrations, 5% HDN exhibited the broadest pore size distribution, ranging from 5 to 150 μm, with 50% of pores falling between 15–30 μm and an average pore size of 46.4 ± 29.1 μm (Figure 4A). In contrast, 10% HDN showed a narrower distribution, with 70% of pores within 15–30 μm and an average pore size of 30.7 ± 12.6 μm (Figure 4B). The 20% HDN cryogels displayed the narrowest distribution, with 85% of pores between 15–30 μm and an average pore size of 25.6 ± 6.3 μm (Figure 4C). These results indicate that higher PEG concentrations lead to smaller pore sizes, consistent with previous studies showing that increasing polymer concentration reduces cryogel pore size.^26^

**Figure 3:**
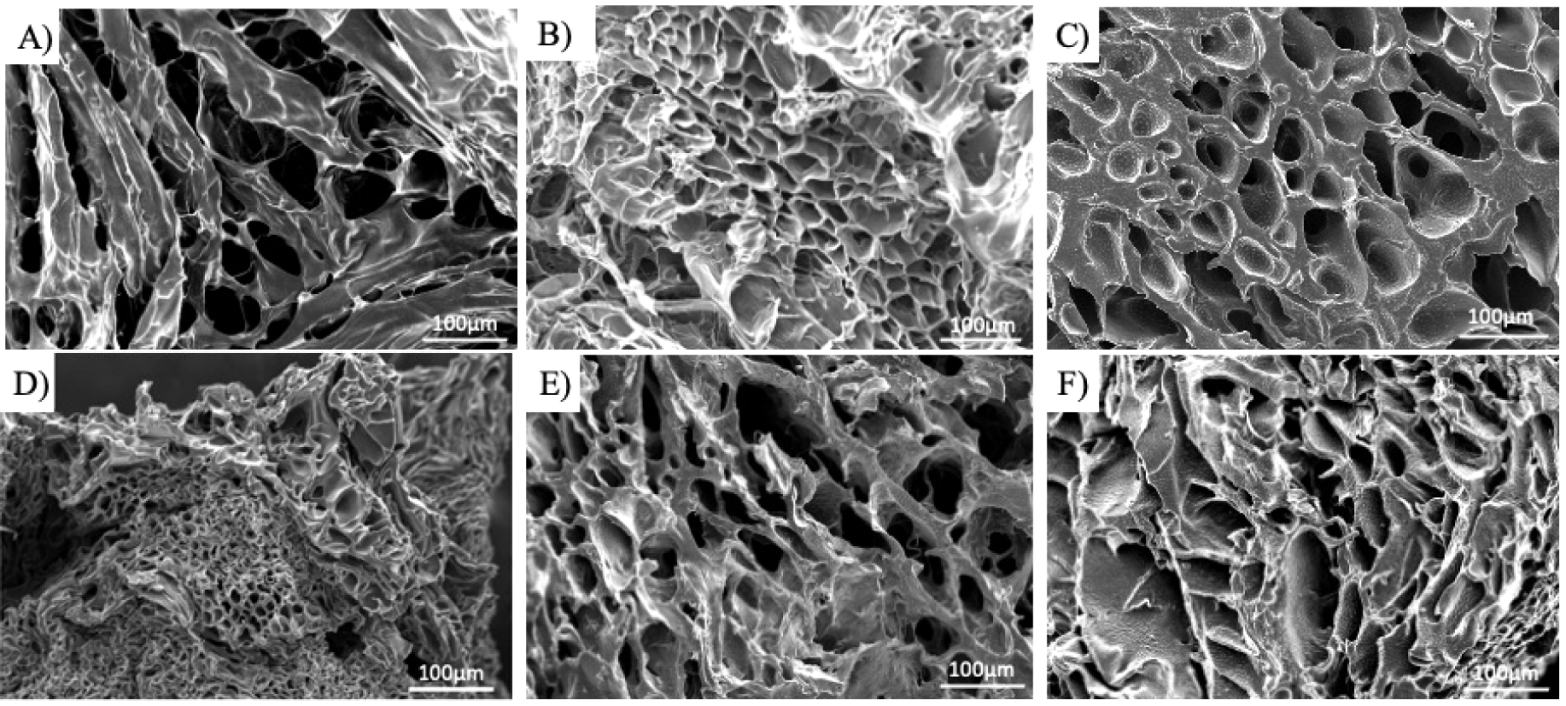
SEM images of PEG-alginate HDN cryogels fabricated by varying synthesis parameters. (A) 5% HDN; (B) 10% HDN; (C) 20% HDN; (D) SA-HDN; (E) 1% AA-HDN; (F) 2% AA-HDN. Scale bar: 100 μm.

**Figure 4:**
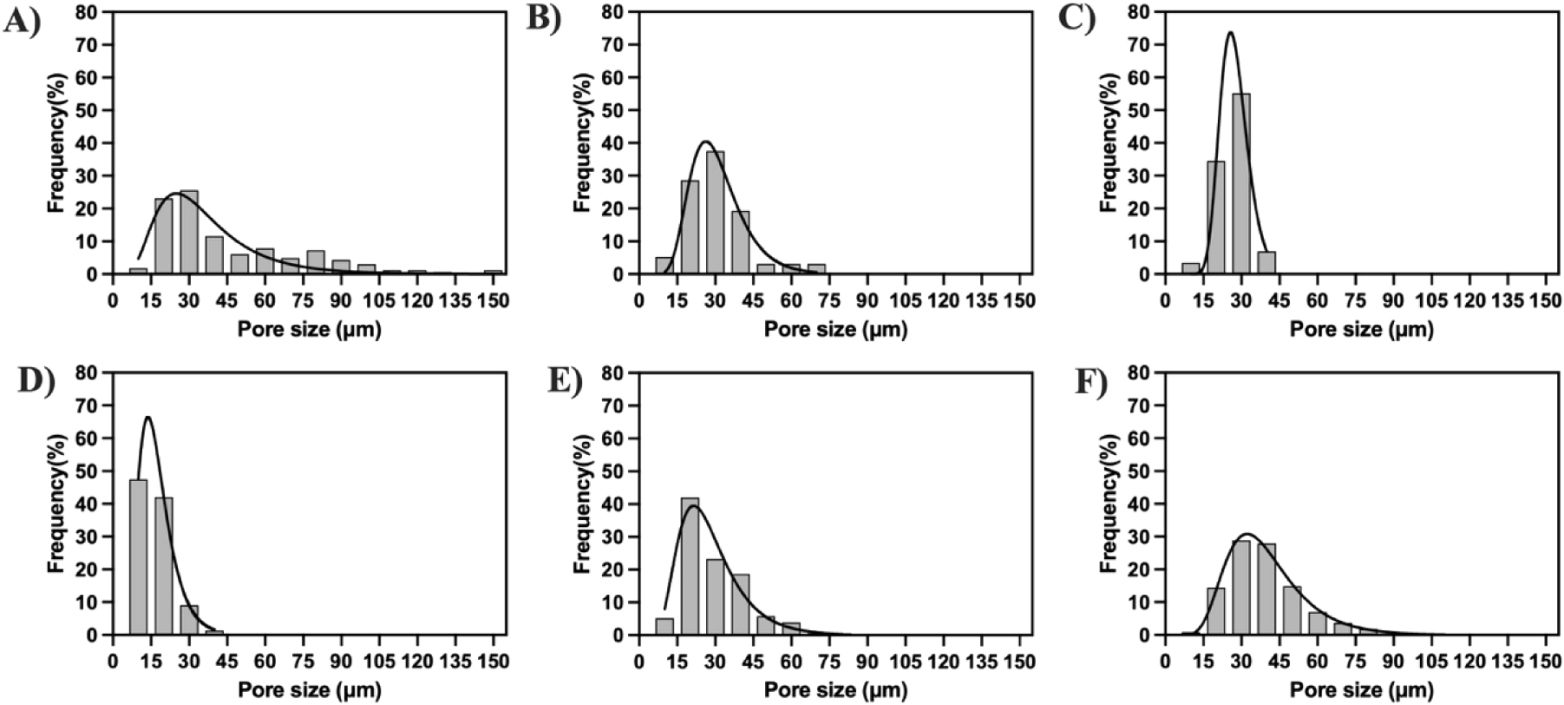
Pore size distribution of HDN cryogels made using varying parameters. Histograms show the frequency percentage (%) of pore size (μm) measured from SEM images acquired at three different regions of each gel. (A) 5% HDN, (B) 10% HDN, (C) 20% HDN, (D) SA-HDN, (E) 1% AA-HDN, and (F) 2% AA-HDN. Black curves represent log-normal distribution fits of the pore size data, overlaid to illustrate the overall distribution trend.

The addition of the ice-nucleating agent l-aspartic acid further increased the pore size and broadened the distribution. Cryogels containing 2% AA-HDN exhibited an average pore size of 38.7 ± 14.7 μm, with ∼50% of pores falling in the 30–45 μm range—larger than that observed in 20% HDN cryogels without l-aspartic acid. This effect can be attributed to the ability of l-aspartic acid to provide nucleation sites, facilitating ice crystal formation at higher subzero temperatures. Due to its zwitterionic structure containing both −NH_3_^+^ and −COO^–^ groups, l-aspartic acid can facilitate directional hydrogen bonding and electrostatic alignment of water molecules to promote heterogeneous ice nucleation and accelerate the freezing process.^47^ The earlier onset of freezing reduces supercooling and allows slower growth of individual ice crystals, resulting in larger pores upon thawing. Although alginate in the formulation also has abundant carboxyl groups, its polymeric structure and high viscosity may suppress nucleation, resulting in less uniformly distributed pores. In contrast, 1% AA-HDN had an average pore size of 29.3 ± 11.8 μm (Figure 4D), which was not significantly different from HDN cryogels, though its distribution extended up to ∼80 μm, indicating a broader spread compared to 20% HDN. Thus, a higher concentration of l-aspartic acid is required to achieve both larger average pore sizes and broader distributions (Figure 4E).^30^ Furthermore, the addition of an ice nucleating agent led to a change in the pore morphology to be more elliptical. This change in pore morphology may be attributed to the changes in the ice crystal size, which may lead to more elliptical pore formation and a change in surface tension experienced as the ice crystals melt away during the thawing phase.

Finally, when cryogels made with unmodified alginate (HDN) were compared to those made with sulfated alginate (SA-HDN), a decrease in pore size was observed. SA-HDN cryogels exhibited an average pore size of 16.7 ± 6.4 μm (Figure 4F), with 95% of pores below 30 μm. Introducing sulfate groups in alginate increases the polyanionic character and hydration of alginate. Although sulfation sometimes reduces direct ice recrystallization inhibition (IRI) ability, our highly hydrated alginate precursor limits mass transport and ice crystal growth, causing smaller and more uniform ice templates during freezing (Figure 3D).^48,49^

This suggests that not only the PEG network polymer concentration but also changes in the chemical structure of the alginate network can impact the formation of ice crystals and pore size. It is worth noting that the pore sizes of the cryogels analyzed in this study were determined from SEM images when the cryogels were in a dry state, which would be smaller than the pore size of the cryogels when they are in the water-absorbing state.^23^

### 3.3 Swelling Kinetics of Cryogels

To evaluate how different parameters influence the swelling behavior of cryogels, we determined equilibrium swelling ratios for all cryogels (Figure 5). HDN cryogels prepared with varying concentrations of multiarm PEG showed distinct swelling ratios. 5% HDN exhibited the highest swelling ratio, reaching ∼1900%, which was nearly twice that of both 10% and 20% HDN. In contrast, no significant difference was observed between 10% and 20% HDN (Figure 5A).

**Figure 5:**
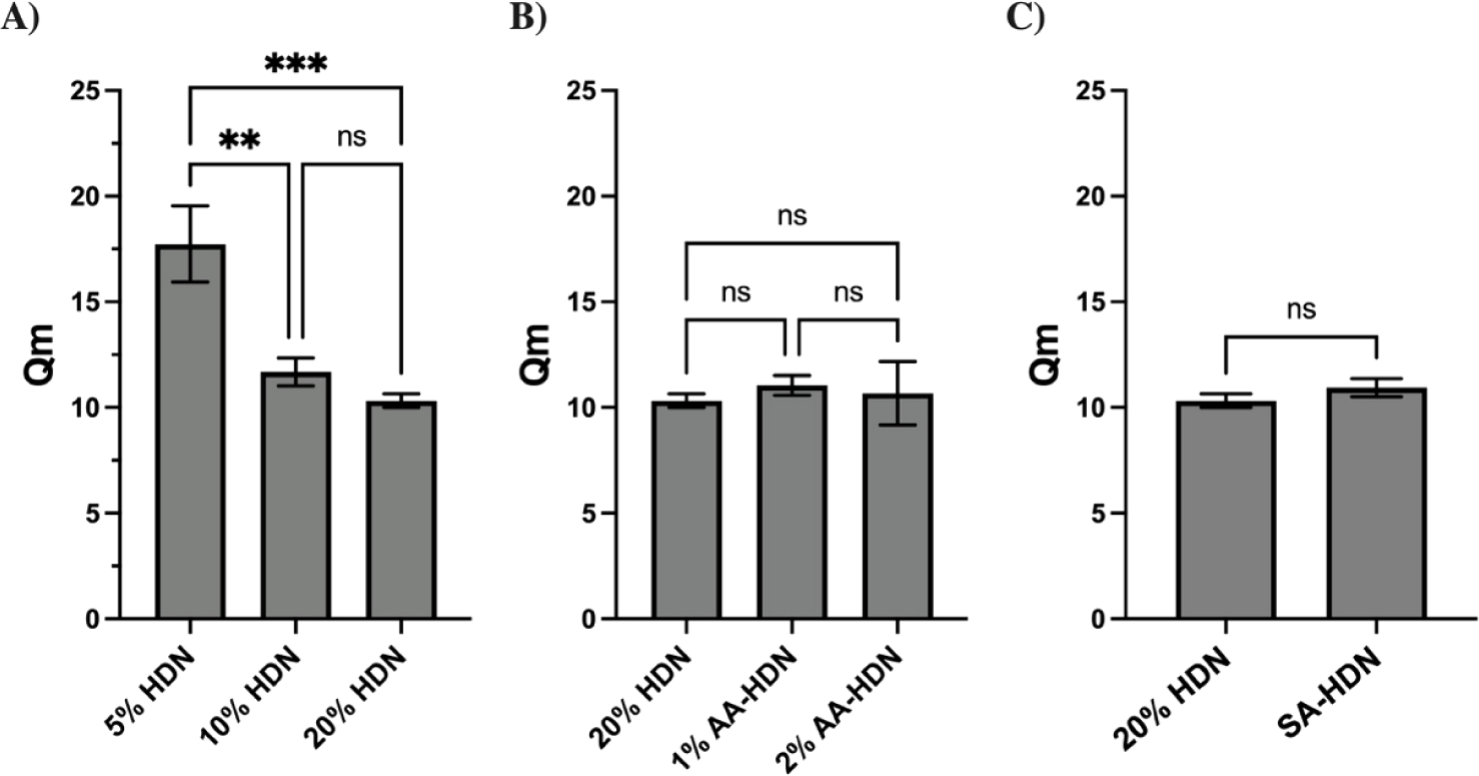
Effect of different parameters on cryogels’ equilibrium swelling ratio: (A) cryogels made with different concentrations of the PEG network: 5% HDN, 10% HDN;, and 20% HDN; (B) cryogels with and without using the ice nucleating agent (l-aspartic acid): 20% HDN (without l-aspartic acid), 1% AA-HDN, and 2%AA-HDN; (C) cryogels made with and without the sulfated alginate network: 20% HDN and SA-HDN. Results are presented as the mean value with its standard deviation. Statistical significance between each group was calculated using one-way ANOVA and *t* test in GraphPad Prism. **** = *P* < 0.0001; *** = *P* < 0.001; ** = *P* < 0.01; * = *P* < 0.05; ns = *P* > 0.05 (*n* ≥ 3).

Cryogels containing l-aspartic acid did not show a significantly higher overall swelling ratio compared with those without it. However, they absorbed water more rapidly, reaching equilibrium swelling within ∼30 min, whereas cryogels without l-aspartic acid required a longer time (Figure 5B).

For SA-HDN cryogels, no significant difference in swelling behavior was observed compared to HDN. Interestingly, despite the reduction in pore size, the sulfated alginate did not alter the overall water uptake capacity of the cryogel system, which can be attributed to the enhanced hydrophilicity and water-binding capacity of sulfated alginate compared to those of unmodified alginate (Figure 5C). Moreover, swelling capacity reflects pore interconnectivity, which may remain unaffected between the two systems.

In terms of the time required to reach equilibrium swelling (Figure 6), 1% AA-HDN equilibrated within ∼5 min, whereas 5% HDN and 2% AA-HDN required ∼1 h. The longest equilibration times (∼2 h) were observed for 10% HDN, 20% HDN, and SA-HDN cryogels. Notably, the rate of equilibration did not correlate with the overall water absorption capacity.

**Figure 6:**
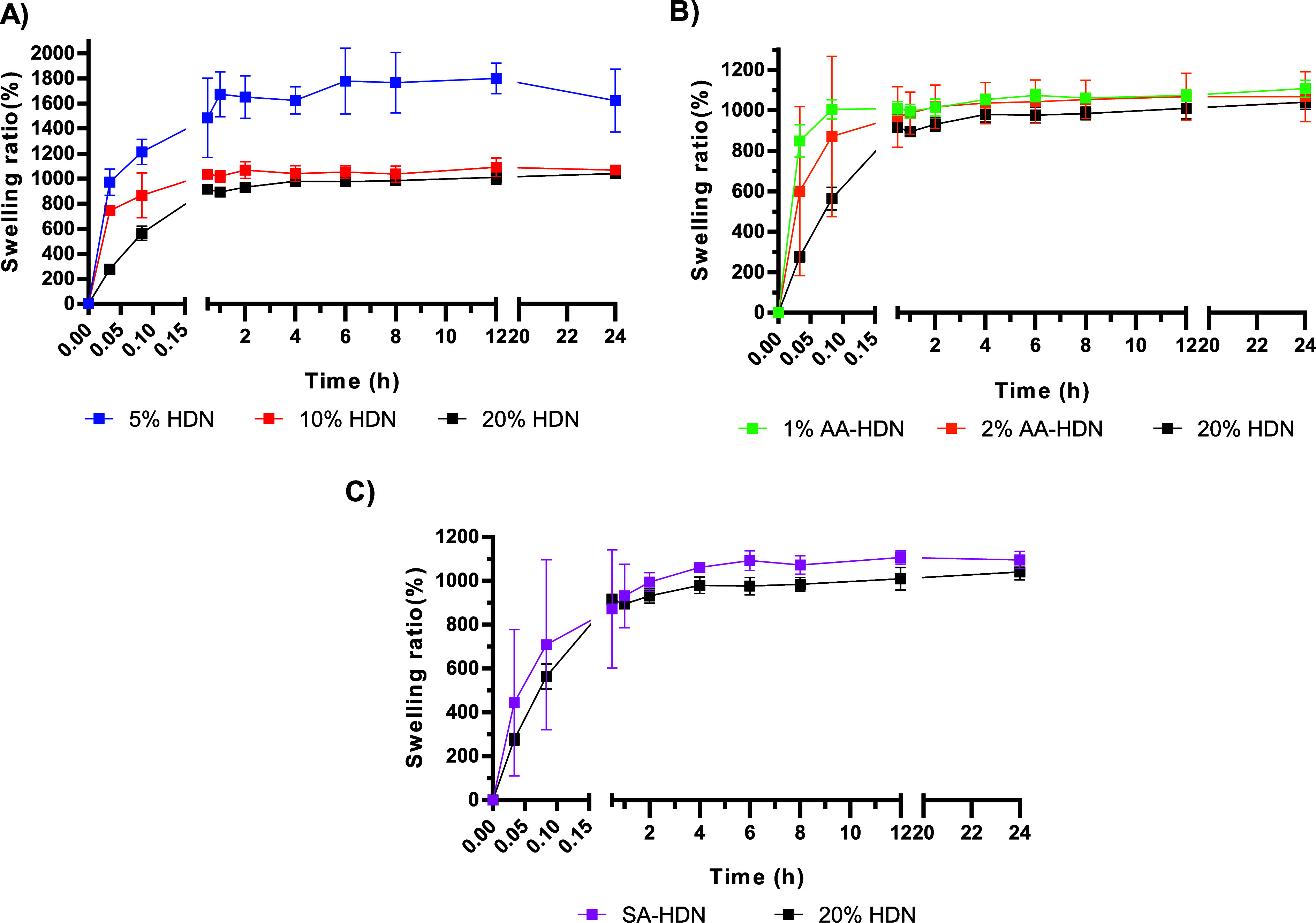
Effect of different parameters on cryogels’ water uptake ability: (A) cryogels made with different concentrations of the PEG network: 5% HDN, 10% HDN, and 20% HDN; (B) cryogels with and without using the ice nucleating agent (l-aspartic acid): 1% AA-HDN, 2%AA-HDN, and 20% HDN (without l-aspartic acid); (C) cryogels made with and without the sulfated alginate network: SA-HDN and 20% HDN. Results are presented as the mean value with its standard deviation (*n* ≥ 3).

The rapid swelling of 1% AA-HDN may be attributed to l-aspartic acid promoting the formation of larger, more interconnected pores, which reduce diffusion resistance and shorten the swelling path. However, increasing the concentration of l-aspartic acid appeared to disrupt network uniformity or enhance polymer–polymer interactions, thereby hindering water penetration and prolonging equilibration.

Despite differences in swelling kinetics, all cryogels demonstrated rapid water uptake, achieving a swelling ratio of ∼800% within the first 5 min. These fast kinetics highlights the efficient transport properties and pore interconnectivity inherent to ice-templated cryogels. Such characteristics are particularly advantageous for tissue engineering applications, where efficient nutrient transport, rapid equilibration with surrounding media, and interconnected pore structures are essential for supporting high-density cell cultures and ensuring long-term cell viability and function.^50,51^

### 3.4 Rheological and Viscoelastic Properties

Viscoelastic materials typically exhibit distinct rheological properties characterized by two regions, storage modulus and loss modulus, which are associated with the elastic and viscous behavior of the material.^52^ At low strain levels, the storage and loss moduli remain unchanged regardless of the strain applied. However, beyond a critical strain level, these moduli gradually decrease as the material displays nonlinear behavior.^53^

Therefore, to determine the storage modulus of the cryogels, we initially conducted a strain amplitude test to identify the linear viscoelastic region of the cryogels (Figure 7A–C). The strain amplitude test was carried out within the range of 0.01% to 1% strain. Figure 7A–C illustrates the results of the strain amplitude tests for all of the cryogels. All cryogels exhibited a stable and similar loss modulus of ∼1.5 kPa within the strain range of 0.01% to 1%.

**Figure 7:**
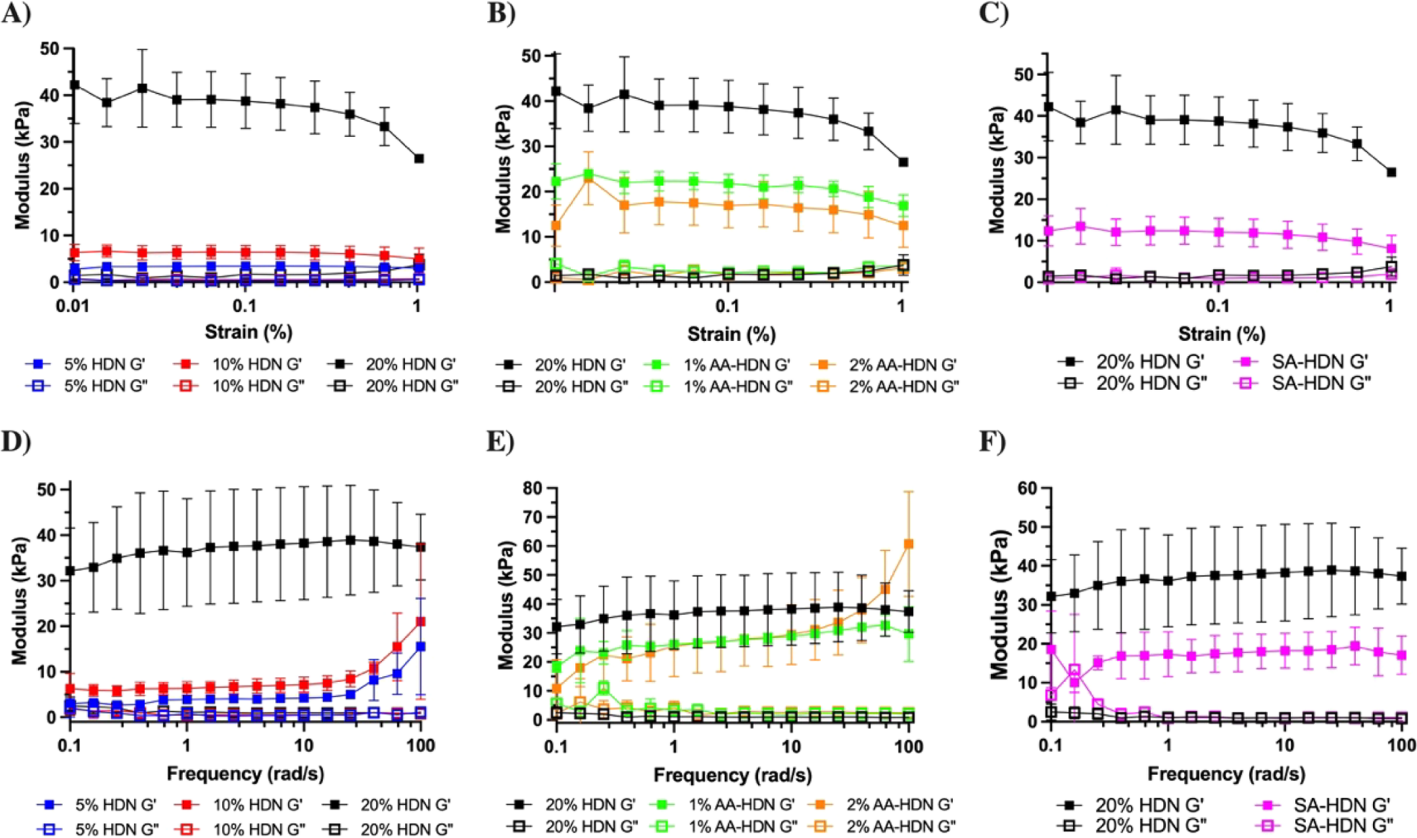
Effect of different parameters on cryogels’ rheological test: (A–C) cryogels’ strain amplitude test; (D, E**)** cryogels’ frequency sweep test; (A, D) cryogels made with different concentrations of the PEG network: 5% HDN, 10% HDN, and 20% HDN; (B, E) cryogels with and without using the ice nucleating agent (l-aspartic acid): 1% AA-HDN, 2%AA-HDN, and 20% HDN (without l-aspartic acid); (C, F) cryogels made with and without the sulfated alginate network: SA-HDN and 20% HDN. Results are presented as the mean value with its standard deviation (*n* ≥ 3).

Within the strain range 0.01% to 0.1%, the modulus of each cryogel exhibited slow and nonlinear changes. As the strain increased to 1%, there was a tendency for all storage moduli to change. Interestingly, prior to the intersection point of the storage modulus and the loss modulus, the increase in strain resulted in a mainly decreasing elastic portion and a slow increase in the viscous portion. This can be attributed to a delay in the structural breakdown during crack propagation.^54^ During this period, the elastic portion still dominated. Once the loss modulus and storage modulus intersected, the viscous portion began to dominate. Consequently, a fixed strain of 0.01% was selected for the frequency amplitude test conducted on all types of cryogels, as this small deformation could simulate the low-strain conditions under most physiological loading.

At a constant strain of 0.01%, we conducted a frequency amplitude test in the frequency range 0.1% to 100% to simulate varying levels of movement intensity (Figure 7 D–E), from weak (low frequency) to powerful (high frequency). For all cryogels, the storage modulus consistently remained higher than the loss modulus, indicating that the materials were stable and behaved as elastic solids. In general, within this range of angular frequencies, 1%AA-HDN, and SA-HDN cryogels exhibited a stable plateau in the storage modulus. The storage modulus of 5% HDN, 10% HDN, and 2% AA-HDN tended to increase as the frequency increased from 1 rad/s to 100 rad/s.

To allow statistical comparison of the storage modulus, a representative storage modulus was obtained from each cryogel at 1 rad/s within the linear viscoelastic region (Figure 8). Overall, 20% HDN cryogels exhibited the highest energy storage capacity under deformation. Reducing the concentration of the PEG network led to a reduction in storage modulus, which was related to a decrease in the number of cross-linking points and polymer concentration (Figure 8A). Although the comparisons were not statistically significant, the average storage modulus showed a decreasing trend when sulfated alginate or l-aspartic acid was incorporated (Figure 8B,C). This may be due to the sulfate group being electronegative, causing electrostatic repulsion with calcium ions, which disrupts the ionic bonding between alginate molecules and weakens the cross-linking strength and density.^55^ Additionally, using the ice nucleating agent l-aspartic acid increased pore size and may have impacted the cross-linking reaction, which caused a weaker gel network and a lower storage modulus.^56–59^

**Figure 8:**
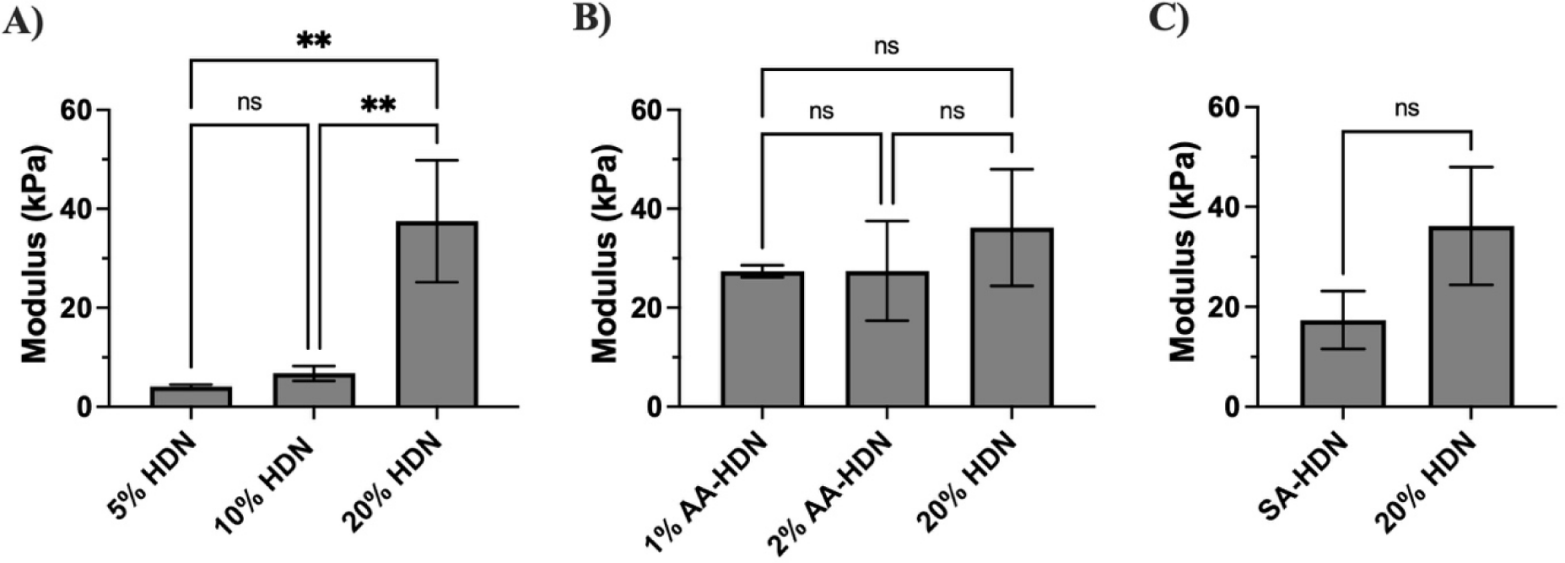
Effect of different parameters on cryogels’ storage modulus. (A) cryogels made with different concentrations on the PEG network: 5% HDN, 10% HDN, and 20% HDN; (B) cryogels with and without using the ice nucleating agent (l-aspartic acid): 1% AA-HDN, 2%AA-HDN, and 20% HDN (without l-aspartic acid); (C) cryogels made with and without the sulfated alginate network: 20% HDN and SA-HDN. Results are presented as the mean value with its standard deviation. Statistical significance between each two groups was calculated using a *t* test in GraphPad. **** = *P* < 0.0001; *** = *P* < 0.001; ** = *P* < 0.01; * = *P* < 0.05; ns = *P* > 0.05 (*n* ≥ 3).

### 3.5 Compression Modulus of Cryogels

Compression tests were conducted to further evaluate the mechanical properties of the cryogels (Figure 9A–C). Although all tests were set to reach 70% strain, some cryogels did not maintain structural integrity up to that level. As expected, 5% HDN cryogels exhibited the lowest mechanical strength; however, none of the samples showed structural breakage, even at 70% strain (Figure 9A), demonstrating their resilience and toughness. With increasing l-aspartic acid concentration, 20% HDN cryogels became less stiff, and no structural failure was observed in 2% AA-HDN even at 40% strain (Figure 9B). Among all groups, SA-HDN cryogels showed the earliest structural failure, breaking at ∼37.5% strain (Figure 9C). By comparison, 20% HDN cryogels failed at ∼39% strain, indicating greater strength and stiffness than SA-HDN, although the difference was not statistically significant.

**Figure 9:**
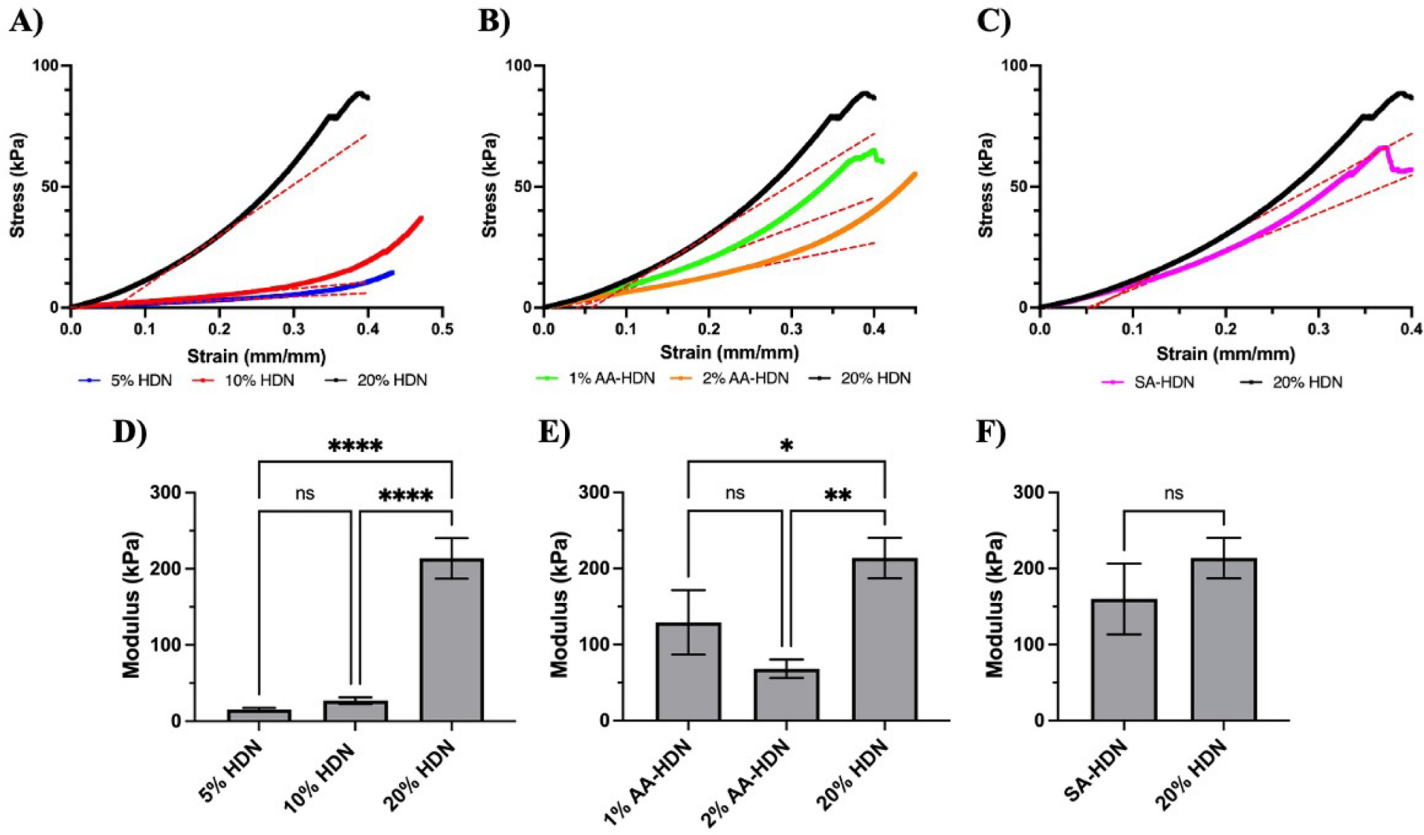
Effect of different parameters on cryogels’ compression modulus: (A–C) cryogels’ strain–stress curve; (D, E) cryogels’ Young’s modulus; (A, D) cryogels made with different concentrations on the PEG network: 5% HDN, 10% HDN, and 20% HDN; (B, E) cryogels with and without using the ice nucleating agent (l-aspartic acid): 1% AA-HDN, 2%AA-HDN, and 20% HDN (without l-aspartic acid); (C, F) cryogels made with and without sulfated alginate: SA-HDN and 20% HDN. Results are presented as the mean value with its standard deviation. Statistical significance between groups was calculated using one-way ANOVA and *t* test in GraphPad Prism. **** = *P* < 0.0001; *** = *P* < 0.001; ** = *P* < 0.01; * = *P* < 0.05; ns = *P* > 0.05 (*n* ≥ 3).

The Young’s modulus, calculated as the slope of the stress–strain curve between 15% and 20% strain (Figure 9D–F), also varied depending on the cryogel composition. The highest modulus was observed for 20% HDN (226.4 kPa), attributable to the formation of a rigid network with high gelation efficiency and strong resistance to deformation. For cryogels with different PEG concentrations (Figure 9D), the modulus increased with increasing polymer concentration, consistent with the expectation that lower cross-linking density at reduced polymer content leads to greater swelling capacity but lower mechanical strength.^56,58^

Cryogels incorporating l-aspartic acid exhibited lower Young’s modulus than their counterparts without l-aspartic acid (Figure 9E), likely due to increased pore size, which weakened the structure and reduced mechanical strength.^56,58,59^ Interestingly, cryogels prepared with sulfated alginate (SA-HDN) showed no significant difference in modulus compared with unmodified alginate (20% HDN) (Figure 9F). This suggests that sulfation did not impair the cryogels’ ability to resist deformation or compromise their compressive strength, despite the smaller pore size observed in SA-HDN.

### 3.6 Release of TGF-β1 and IGF-1 from Cryogels In Vitro

To evaluate the suitability of cryogels as potential scaffolds for tissue engineering, we assessed their ability to continuously release key growth factors necessary for tissue regeneration. TGF-β1 and IGF-1 were loaded into HDN and SA-HDN cryogels, and their release profiles were studied over 7 days (Figure 10). The final loading levels of TGF-β1 and IGF-1 in HDN cryogels were 95 ± 1.8% and 99 ± 0.1%, respectively. For SA-HDN cryogels, the final loading levels were 96 ± 0.9% for TGF-β1 and 99 ± 0.5% for IGF-1. This indicates that both HDN and SA-HDN cryogels had a loading efficiency of over 95% for both growth factors with no significant difference between them. TGF-β1 was continuously released from both cryogels over the seven-day period. In HDN cryogels, about 1% was released in the first hour, and by day 7, approximately 7% was released. In SA-HDN cryogels, about 0.8% was released in the first hour, with around 4.9% released by day 7. Compared with HDN, SA-HDN cryogels released less TGF-β1. IGF-1 was continuously released from HDN cryogels over 2 days and from SA-HDN cryogels over 3 days. For HDN cryogels, about 0.5% was released in the first hour and about 1.5% by day 2. In SA-HDN cryogels, about 0.4% was released in the first hour, with approximately 1.7% released by day 3. The total amount of IGF-1 released from both types of cryogels was similar. The differences in the release kinetics of TGF-β1 and IGF-1 can be attributed to their molecular weights and affinities for the cryogel matrix. TGF-β1, a 25 kDa protein, has a molecular weight of approximately three times that of IGF-1 (7.6 kDa). Additionally, charged interactions between alginate and growth factors may slow their diffusion from the macroporous cryogels. Moreover, the high retention ability in both systems shows that the double network structure promotes growth factor immobilization irrespective of the presence of sulfated alginate.

**Figure 10:**
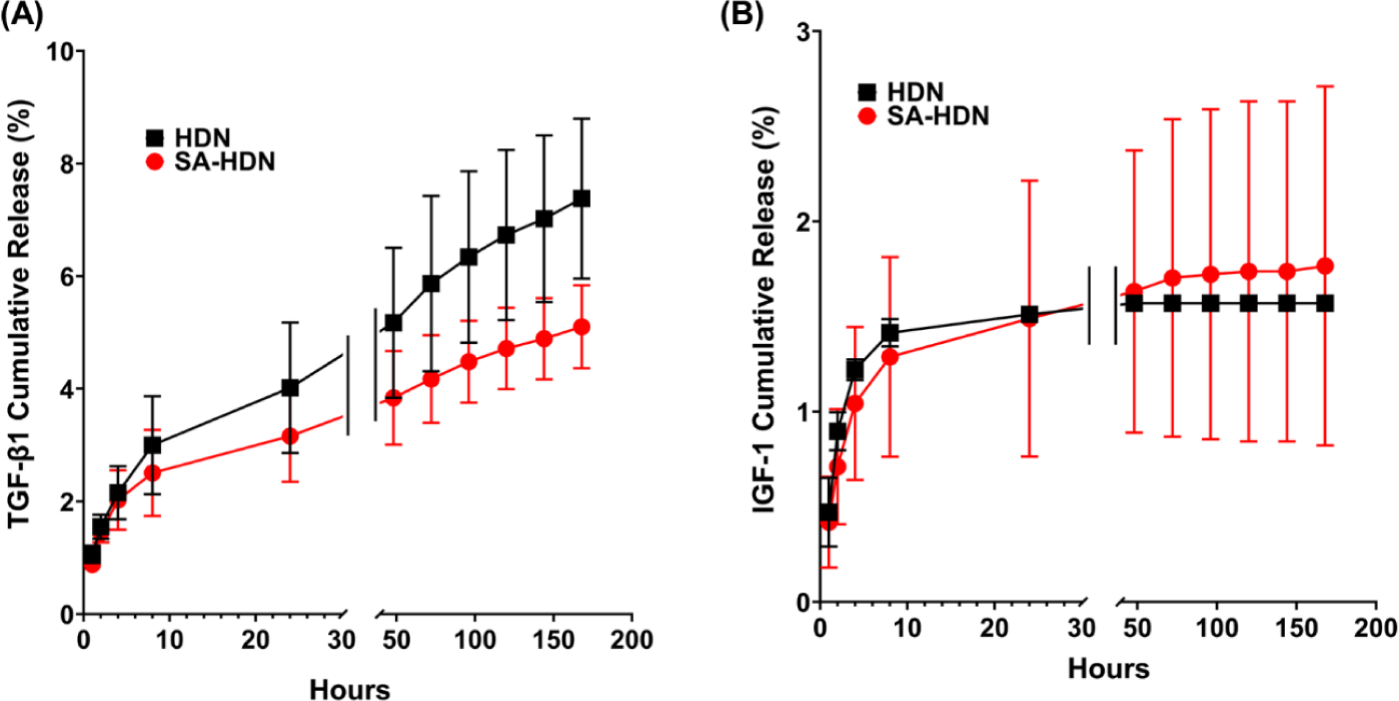
Percentage cumulative release of growth factors: (A) TGF-β1 and (B) IGF-1 from HDN and SA-HDN cryogels. Percentage of growth factors released daily was measured by ELISA until 7 days. Cumulative release (%) was calculated by adding daily release values divided by the loading mass. Results are presented as the mean value with standard deviation (*n* ≥ 3).

It is noticeable that TGF-β1 is released faster than IGF-1. This indicates the process of diffusion, driven by the molecular weight and electrostatic attraction between the negatively charged alginate chains and the positively charged protein domains. In addition to the above-mentioned interactions, IGF-1 showed approximately 98% binding, indicating a higher level of interaction between IGF-1 through hydrogen and ionic bonds arising from the dual network. All of the above indicate that the interactions between polymer and proteins may include physical retention, electrostatic, and hydrogen-bond interactions. These findings suggest that the HDN and SA-HDN cryogels prepared in this study have high potential for the sustained delivery of growth factors that are critical for tissue regeneration.

Further analysis of the growth factor release kinetics using multiple mathematical models revealed that the Korsmeyer–Peppas (KP) model provided the best overall fit to the data (Figures S2A and 3A). The KP model yielded the highest *R* ^2^ values for both HDN and SA-HDN cryogels, with *R* ^2^ > 0.92, indicating a strong correlation and robust predictive capability (Table S1). For the HDN cryogels, the TGFβ1 release profile exhibited an excellent fit (*R* ^2^ = 0.997), surpassing that of IGF-1 (*R* ^2^ = 0.857), suggesting distinct release behaviors for the two growth factors. The lower *R* ^2^ observed for IGF-1 may indicate a higher degree of retention within the HDN matrix or the involvement of additional release mechanisms beyond diffusion such as polymer relaxation or matrix swelling.

In contrast, release data from SA-HDN cryogels showed consistently high KP model fits for both growth factors (*R* ^2^ > 0.94), further supporting the applicability of this model to the cryogel system. Based on the KP analysis, the diffusional exponent *n* < 0.45 for all conditions, indicating a quasi-Fickian diffusion mechanism, where diffusion remains the dominant release pathway with contributions from polymer swelling or slow erosion. This hybrid mechanism aligns well with the structural characteristics of cryogels, whose highly interconnected macroporous architecture facilitates water uptake, swelling, and gradual matrix relaxation over time.

Fitting the data to the Higuchi model produced an *R* ^2^ of 0.97 for TGFβ1 release, which—although high—was still lower than that of the KP model and consistent with visible deviations between experimental and fitted curves (Figure S2D). The Higuchi fit for IGF-1 was substantially weaker (*R* ^2^ = 0.74–0.86), further indicating that classical square-root diffusion does not fully capture the release behavior of this growth factor (Figure S3D). Both the first-order and zero-order models provided comparatively poor fits (*R* ^2^ = 0.62–0.92), demonstrating limited compatibility with the experimentally observed release kinetics (Figures S2 and S3B,C).

Collectively, these results demonstrate that the KP model most accurately describes the release of TGFβ1 and IGF-1 from both HDN and SA-HDN cryogels, supporting a mechanism governed primarily by diffusion, with secondary contributions from polymer swelling or relaxation. This mixed-mode release behavior is consistent with the physicochemical characteristics of cryogels, whose macroporous network swells and undergoes gradual structural changes during prolonged exposure to aqueous environments. Representative model-fit graphs and summarized kinetic parameters (k, n, and R^2^) are provided in the Supporting Information.

### 3.7 Biocompatibility of Cryogels

To assess the biocompatibility of HDN cryogels, clonally derived mouse D1MSCs were seeded into collagen I-coated cryogels (50 μg/mL). Three representative formulations—20% HDN, 2% AA-HDN, and SA-HDN—were selected to evaluate the effects of polymer concentration, ice nucleator addition, and alginate sulfation, respectively. Cell viability was analyzed on days 3 and 7 using LIVE/DEAD staining. As shown in Figure 11A, cells remained viable and were uniformly distributed throughout the cryogels. On day 3, cell viability was 65.7% in 20% HDN, compared to 74.6% in 2% AA-HDN and 80.9% in SA-HDN (Figure 11B). By day 7, viability declined to 56.8% in 20% HDN but remained stable at 75.2% in 2% AA-HDN and increased further to 91.2% in SA-HDN. Statistical analysis revealed no significant differences among groups on day 3, while on day 7, only SA-HDN exhibited significantly higher viability compared to 20% HDN. According to ISO 10993-5-2009 standards, both 2% AA-HDN and SA-HDN demonstrated excellent biocompatibility with no evidence of cytotoxicity.

**Figure 11:**
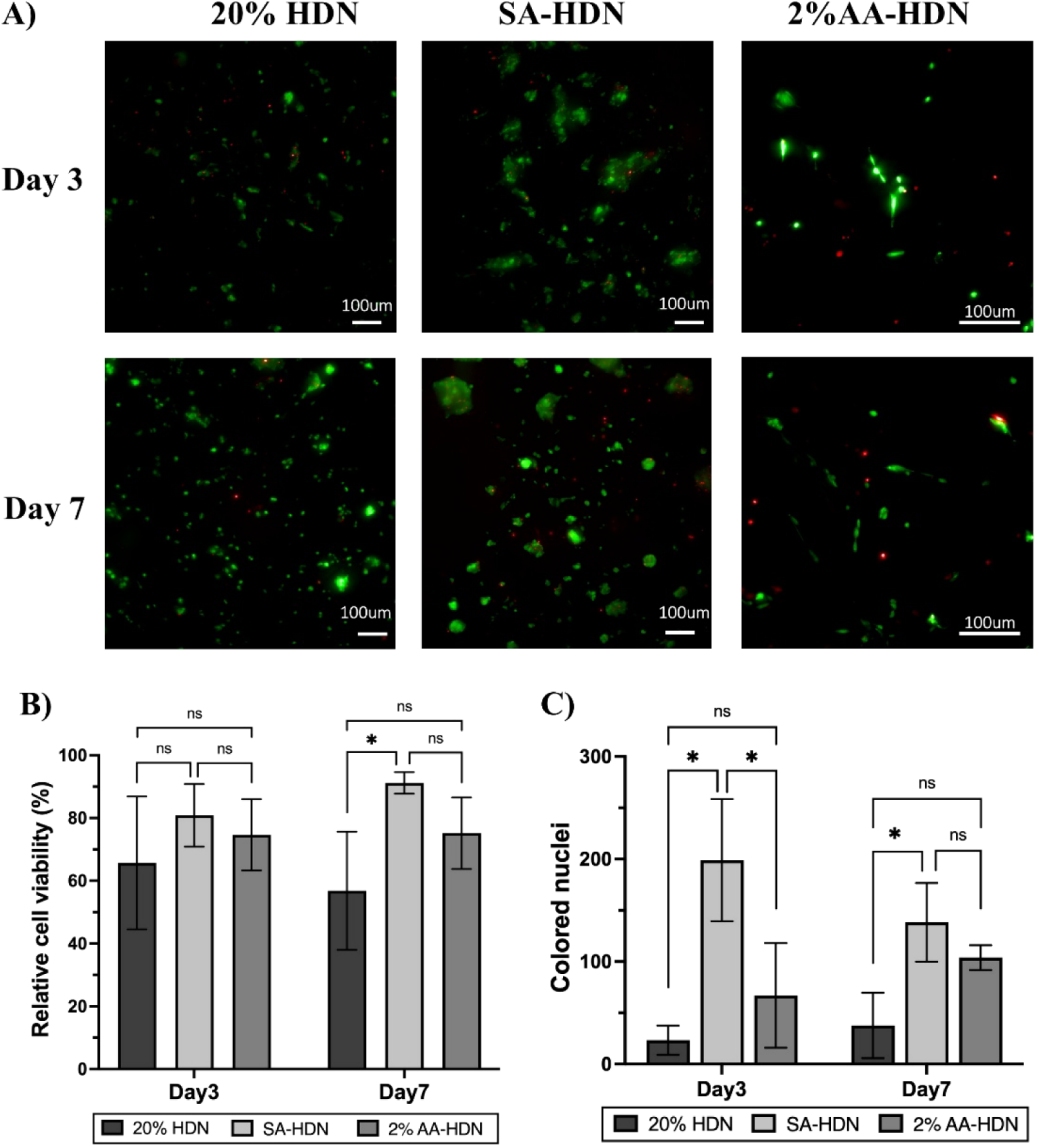
Biocompatibility for cryogels: (A) LIVE/DEAD staining images of D1MSCs cultured in 20% HDN, SA-HDN, and 2%AA-HDN for 3 and 7 days. (B) Cell viability (%) after culture in the HDN cryogels after 3 and 7 days. (C) Statistical analysis of DAPI Stained Colored nuclei of D1MSCs cultured in HDN cryogels at days 3 and 7. Results are presented as the mean value with its standard deviation. Statistical significance between each two groups was calculated using *t* test in GraphPad Prism. **** = *P* < 0.0001; *** = *P* < 0.001; ** = *P* < 0.01; * = *P* < 0.05; ns = *P* > 0.05 (*n* ≥ 3).

The superior performance of SA-HDN can be attributed to its similarity to native ECM and the presence of charge (16.7 ± 6.4 μm), which may better support cell–matrix interactions and facilitate cell attachment and proliferation. By contrast, the denser network structure of 20% HDN likely restricted nutrient diffusion and limited space for cell expansion, resulting in reduced viability over time. 2% AA-HDN, with its larger and more interconnected pores, supported stable viability, suggesting that using an ice nucleating agent, l-aspartic acid, improved the pore microarchitecture of 20% HDN cryogels for maintaining cells but did not enhance proliferation as effectively as sulfation. Thus, this indicates that a combination of these strategies may be needed in the future to obtain cryogels with optimum pore size and biocompatibility. DAPI staining corroborated these findings: the fewest nuclei were observed in 20% HDN, while SA-HDN showed the highest number of nuclei, indicating enhanced proliferation (Figure 11C). Taken together, these results highlight that cryogel pore size and chemical modifications strongly influence cell survival and proliferation, with sulfation of alginate providing the most favorable microenvironment for MSC culture.

## 4 Conclusion

In this study, we developed a series of biocompatible hybrid double-network (HDN) cryogels and systematically examined how the polymer concentration, incorporation of an ice-nucleating agent, and chemical modification of alginate influence cryogel properties. We demonstrated that increasing polymer concentration enhances mechanical strength, whereas the addition of l-aspartic acid enlarges pore size but reduces stiffness and alginate sulfation decreases mechanical strength despite improving biocompatibility. We did not evaluate the synergistic effect of these three different parameters on the cryogel structure, as the aim here was to establish how each parameter affected cryogel properties compared to our baseline formulation 20%HDN. Nonetheless, these findings underscore the critical role of both physical and chemical parameters in tailoring cryogels for biomedical applications. Future studies will combine these optimized modifications to construct a structurally and functionally enhanced HDN cryogel system.

Functional evaluations confirmed that the cryogels supported MSC survival and proliferation and enabled controlled growth factor release, highlighting their potential as scaffolds for load-bearing soft tissue regeneration.^60,61^ The HDN cryogels reached a compressive modulus of ∼226 kPa and a storage modulus of 36.8 ± 9.7 kPa, values that fall within the physiological range of load-bearing tissues such as cartilage, meniscus, and intervertebral disc. This suggests that the cryogels can be adapted to address the mechanically demanding soft tissue engineering needs, where both mechanical resilience and porosity are important. Looking forward, optimizing injectability without compromising mechanical performance will be essential for minimally invasive delivery into focal tissue defects. Furthermore, evaluating the stability of HDN cryogels at the defect site will be crucial to ensure adequate extracellular matrix deposition and tissue formation before scaffold degradation.

Overall, our findings demonstrate that HDN cryogels combine high porosity, favorable swelling, and appropriate mechanical strength, making them a promising scaffold for load-bearing soft tissue. By bridging the gap between cell-friendly matrices and mechanically robust materials, these cryogels represent a versatile platform for engineering functional tissue. Future studies will focus on evaluating their performance in preclinical models and optimizing injectability for minimally invasive applications.

## Supplementary Material



## Notes

Figure S1: SEM images of HDN cryogels showing the effect of l-aspartic acid (Asp) as an ice nucleating agent on pore morphology; Figure S2: Analysis and model fitting of kinetic release data for TGFβ-1 release from HDN-20% and SA-HDN cryogels; Figure S3: Analysis and model fitting of kinetic release data for IGF-1 release from HDN-20% and SA-HDN cryogels; Table S1: Analysis and model fitting of kinetic release data for TGF-β and IGF-1 release from HDN-20% and SA-HD cryogels (PDF)

## #

Department of Bioengineering, University of Texas at Arlington, Arlington, TX 76010, USA
