# Supplementary material for: Macroporous Alginate–PEG Hybrid Double Network Cryogels: Tuning Mechanics, Porosity, and Long-Term Growth Factor Release via Polymer Concentration, Ice Nucleation, and Sulfation
Source: ACS Appl Bio Mater. 2025 Dec 27;9(2):1039–52. doi: 10.1021/acsabm.5c01929 (PMC12820959; doi:10.1021/acsabm.5c01929)
Supplement: Supplementary file 1 [file mt5c01929_si_001.pdf]

Supplementary Information

Macroporous Alginate–PEG Hybrid Double Network Cryogels: Tuning Mechanics, Porosity, and Long-Term Growth Factor Release via Polymer Concentration, Ice Nucleation, and Sulfation

*Zining Yang1,2& #, Kaixiang Zhang1,2&, Michael Patrick Seitz1,2, and Era Jain, PhD1,2**

1 Department of Biomedical and Chemical engineering,2 Bioinspired Syracuse: Institute for Material and Living System, Syracuse University, Syracuse, NY13244, USA

kzhang57@syr.edu, zxy9464@mavs.uta.edu, mseitz@syr.edu, erjain@syr.edu

***Corresponding Author**

Era Jain (Ph.D.)
Biomedical and Chemical Engineering
Bioinspired Syracuse: Institute for Material and Living System
Syracuse University
Syracuse, NY, USA, 13244
Tel: 315.443.4050
Email: erjain@syr.edu

& denotes equal authorship

# Present Address: Department of Bioengineering, University of Texas at Arlington, Arlington, TX 76010, USA

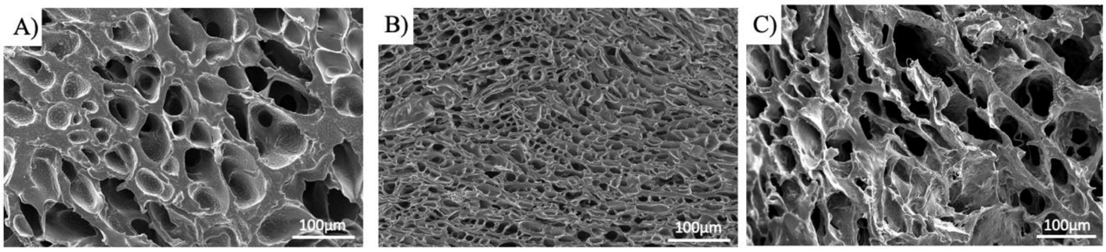

Figure S1: SEM images of HDN cryogels showing the effect of L-aspartic acid (Asp) as an ice nucleating agent on pore morphology. (A) 20% HDN; (B) 20% HDN containing 1% Asp, prepared at −20 °C; (C) 1% AA-HDN (20% HDN + 1% Asp), prepared at −12 °C. Scale bar: 100 µm.

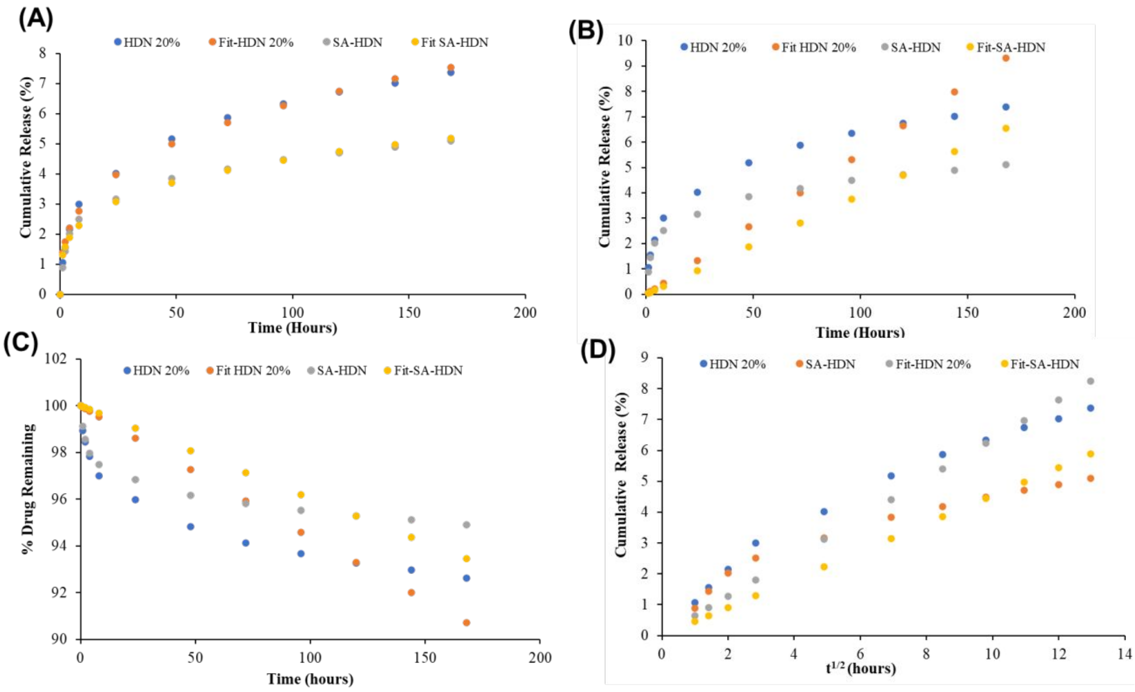

Figure S2: Analysis and model fitting of kinetic release data for TGFβ-1 release from HDN-20% and SA-HDN cryogels: A) Korsmeyer-Peppas model B) Zero-order; C) First-order and D) Higuchi model.

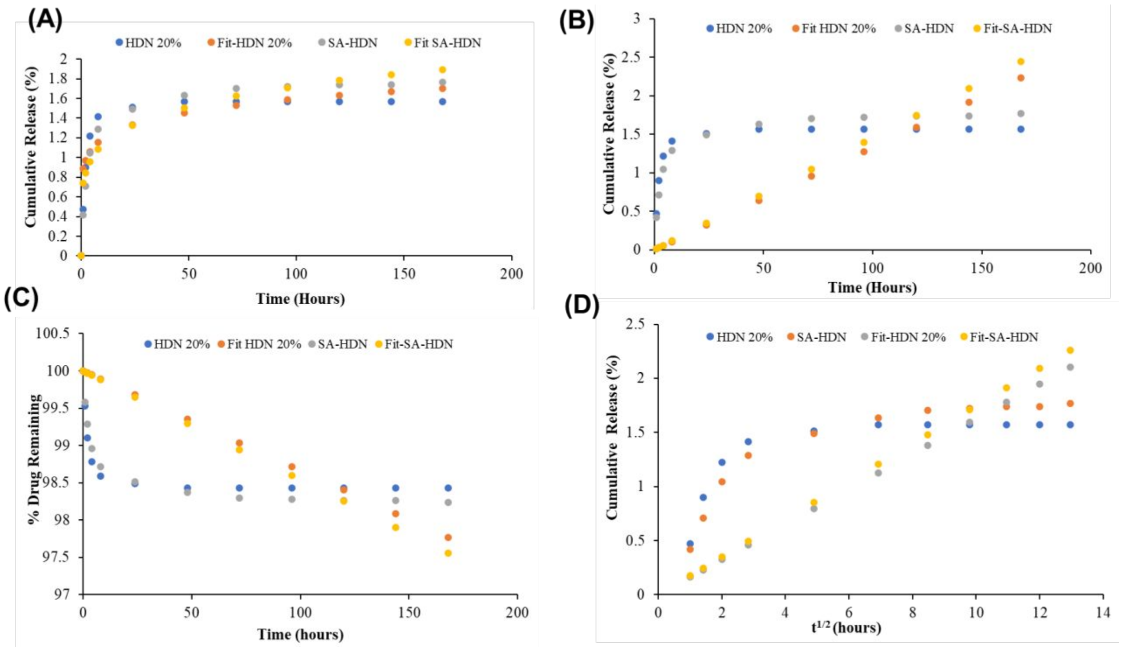

Figure S3: Analysis and model fitting of kinetic release data for IGF-1 release from HDN-20% and SA-HDN cryogels: A) Korsmeyer-Peppas model B) Zero-order; C) First-order and D) Higuchi model.

Table S1: Analysis and model fitting of kinetic release data for TGF-β and IGF-1 release from HDN-20% and SA-HD cryogels. Correlation coefficient (R^2), K and n values for Korsmeyer-Peppas model (KP), Zero-order, First-order, Higuchi model.

**Sample**		**KP**	**Zero Order**	**First Order**	**Higuchi**
**TGFβ1-HDN 20%**	K	1.396	0.055	0.000579	0.636
	n	0.328			
	R^2	0.997	0.932	0.919	0.97
**TGF β1-SA-HDN**	K	1.304	0.039	0.0004	0.45
	n	0.269			
	R^2	0.995	0.882	0.885	0.973
**IGF-1-HDN**	K	0.889	0.0132	0.000134	0.162
	n	0.126			
	R^2	0.857	0.625	0.617	0.747
**IGF-1 SA-HDN**	K	0.741	0.0145	0.00014	0.174
	n	0.183			
	R^2	0.942	0.768	0.774	0.868
